# Case report: Use of sotatercept to treat pulmonary arterial hypertension in a patient with hereditary hemorrhagic telangiectasia

**DOI:** 10.1016/j.rmcr.2025.102291

**Published:** 2025-09-16

**Authors:** Abinaya Ramakrishnan, Scott Olitsky, Rajan Saggar, Richard N. Channick, Justin P. McWilliams

**Affiliations:** a Division of Interventional Radiology, Department of Radiology, UCLA Medical Center, David Geffen School of Medicine at UCLA, Los Angeles, CA, USA; b CureHHT, HHT Foundation International, Monkton, MD, USA; c Pulmonary Vascular Disease Program, Division of Pulmonary, Critical Care, and Sleep Medicine, UCLA Medical Center, David Geffen School of Medicine at UCLA, Los Angeles, CA, USA

**Keywords:** Hereditary hemorrhagic telangiectasia, Pulmonary arterial hypertension, Sotatercept, Epistaxis

## Abstract

This case report describes a 45-year-old female with HHT type 1 (ENG mutation) and congenital heart disease (sinus venosus atrial septal defect and partial anomalous pulmonary venous return), diagnosed with severe PAH at age 16. Despite long-term treatment with epoprostenol, treprostinil, macitentan, and tadalafil, her condition progressed, leading to evaluation for lung transplantation. In May 2024, sotatercept, a novel TGF-β superfamily ligand trap, was initiated, resulting in significant improvement in exertional capacity and quality of life. The patient experienced increased epistaxis frequency (2–4 episodes/week), though hemoglobin levels rose from 13.0 to 15.4 g/dL. No new telangiectasias or other adverse events were noted. This is the first reported case of sotatercept use in PAH with HHT, highlighting its potential efficacy but also the need for careful monitoring due to increased bleeding risk.

## Article Highlights

• This is the first known case report of sotatercept use in a patient with genetically confirmed HHT and PAH.
• Sotatercept improved functional status and quality of life despite an increase in epistaxis frequency.
• The case underscores the need to weigh bleeding risk against PAH benefit when using sotatercept in patients with HHT.

## 1 Introduction

Hereditary hemorrhagic telangiectasia (HHT) is an autosomal dominant condition with an estimated worldwide prevalence approximately 1 in 5000 individuals [1]. Diagnosis is based on genetic testing or on the Curacao criteria that include (1) recurrent and spontaneous epistaxis; (2) visceral arteriovenous malformations; (3) an affected first-degree family member; and (4) the presence of mucocutaneous telangiectasias. HHT may cause visceral arteriovenous malformations (AVMs), particularly in the lungs, liver, and central venous system, that create high flow channels and are susceptible to rupture and hemorrhage [2,3]. HHT can also be complicated by pulmonary hypertension (PH). PH in HHT is often related to high cardiac output in the setting of diffuse hepatic AVMs, but a small number of HHT patients will develop pulmonary arterial hypertension (PAH) which mimics idiopathic PAH [4,5].

The current treatment regimen for HHT patients with PAH involves the use of targeted therapies, including endothelin receptor antagonists, phosphodiesterase inhibitors, soluble guanylate cyclase stimulators, and prostacyclin pathway agents [[6], [7], [8], [9], [10], [11], [12]]. Despite benefit of these therapies in HHT-associated PAH, there are unique concerns in patients with HHT. For example, endothelin receptor antagonists and soluble guanylate cyclase stimulators may increase the risk of anemia [13,14], phosphodiesterase inhibitors may increase rates of epistaxis and GI bleeding, and prostanoids possess antiplatelet properties [15].

In this context, sotatercept, a first-in-class fusion protein, emerges as a potential therapeutic option, offering a novel approach to manage PAH. Sotatercept works by sequestering specific ligands in the TGF-β superfamily, and restoring pulmonary vascular homeostasis through growth-inhibiting and proapoptotic signaling. In the phase 2 PULSAR trial and phase 3 STELLAR trial, sotatercept was shown to significantly improve the six-minute walk distance, World Health Organization functional class, and pulmonary vascular resistance in patients with PAH receiving background therapy [16,17]. Despite its proven efficacy, adverse events including epistaxis and telangiectasia development could limit sotatercept's use in HHT-PAH. Here, we present a case report of a patient with treatment-resistant PAH who was treated with sotatercept. To our knowledge, this is the first case report documenting the outcomes of sotatercept use in an HHT patient, and we believe it may broaden treatment options for HHT patients with treatment-resistant PAH.

## 2 Case presentation

**Fig. 1:**
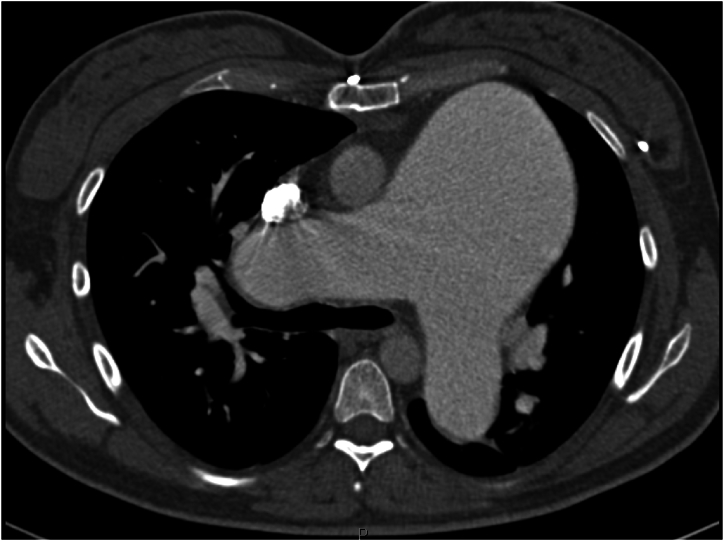
Coronal Computed Tomography (CT) scan of the chest taken in 1997. The image reveals an enlarged main pulmonary artery consistent with severe PAH.

**Fig. 2:**
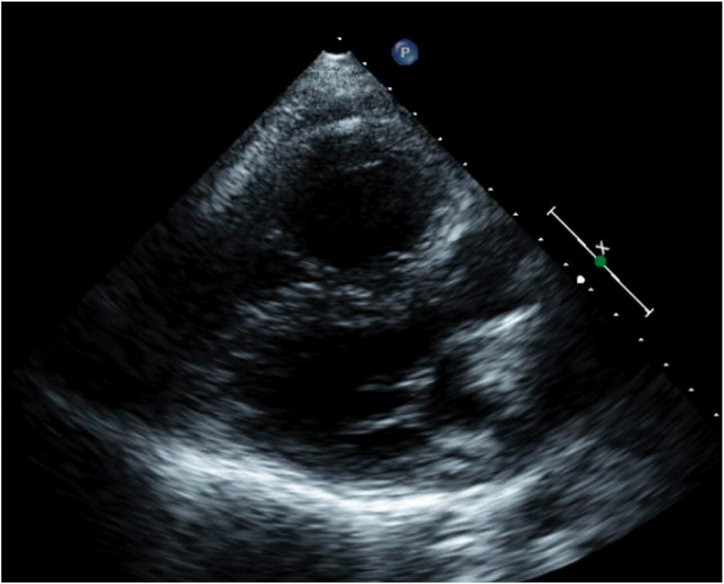
Adult congenital echocardiogram from 2009. Right ventricular enlargement and hypertrophy (RVOT diameter at SAX AV = 31mm; RVIT Apex to basal lateral TV annulus and SAX @ TV = 66 × 41mm), TV DTI SA = 7 cm/s and RVEF = 40 %. Markedly dilated main pulmonary artery (43 mm). RVOT acceleration time = 56 msec consistent with elevated mean pulmonary artery pressure of 54 mmHg.

A 45-year-old female presented with PAH diagnosed at age 16 (1996), when she experienced significant dyspnea during the first trimester of pregnancy. The workup revealed a sinus venosus atrial septal defect (ASD), partial anomalous pulmonary venous return (PAPVR) [right superior pulmonary vein], patent foramen ovale, and a right-to-left shunt. She became oxygen-dependent and underwent termination of pregnancy at 5 months. A right heart catheterization confirmed severe PAH, and she was started on continuous intravenous epoprostenol (Flolan) infusion in 1997, which improved her symptoms and allowed her to return to work. Fig. 1 (Computed Tomography (CT) scan) and Fig. 2 (adult congenital echocardiogram) demonstrate the significant enlargement of the pulmonary artery.

Over the next year, epoprostenol therapy resulted in transition to left-to-right shunt (Qp/Qs 1.5) with the following hemodynamics: right atrial pressure 14 mmHg; pulmonary artery pressure 103/47/67 mmHg; pulmonary artery wedge pressure 14 mmHg; pulmonary blood flow 3.54 L/minute; PVR 15 Wood Units; systemic blood pressure 102/62/75; pulmonary artery oxygen saturation 83 %; radial artery oxygen saturation 99 %. Epoprostenol treatment also led to resolution of hypoxemia, and therefore, she underwent surgical repair including closure of the sinus venosus ASD and patent foramen ovale, a pericardial patch to baffle the anomalous pulmonary vein to the left atrium, and tricuspid and pulmonary valve annuloplasty in 1998. Eventually, due to her slowly progressive functional limitation and compromised quality of life, she was evaluated and approved for lung transplantation in 2021.

In May 2024, she initiated sotatercept (added to background intravenous treprostinil, macitentan, and tadalafil) and was able to uptitrate to the target dose (0.7mg/kg). After 5 doses of sotatercept, the patient noted significant improvement in her exertional limitation and quality of life. She did notice new daily epistaxis, but without decline in hemoglobin, rash, headache, telangiectasia, or ecchymosis. She was referred to the UCLA HHT treatment center for possible nasal cauterization, a procedure she had previously undergone during her adolescent years.

On evaluation, genetic testing revealed a heterozygous *ENG* deletion (exon 9–10), indicating a pathogenic variant associated with HHT type 1. In retrospect, the patient reported a history of recurrent epistaxis since childhood, which had decreased in frequency during the course of her pulmonary arterial hypertension (PAH) from 1997 to 2024. During that period, she experienced approximately 3–4 episodes of epistaxis per year, each lasting no more than 5 min. Following the initiation of sotatercept therapy, she noted an increase in epistaxis frequency to 2–4 episodes per week, with durations ranging from 5 to 30 minutes (average of 10 minutes). These episodes usually occurred in the morning and were variably described as dripping or gushing. The severity of epistaxis was moderate, with an epistaxis severity score of 4.2 [18]. Family history was notable for a paternal uncle with clinically diagnosed HHT. Physical exam while on sotatercept was notable for normal work of breathing, with normal oxygen saturation at rest and exertion. There were no lung or liver AVMs present on chest and abdominal CT scans, respectively. There were no significant changes in platelet counts during sotatercept therapy, however there was an increase in average hemoglobin from 13.0 prior to sotatercept to 15.4 after starting sotatercept. We hypothesize that the rise in hemoglobin despite increased epistaxis reflects sotatercept's erythropoietic effects, which outweighed the patient's blood loss from mucosal bleeding [21,22]. The patient described the epistaxis as bothersome, but “well worth it" given her significant clinical improvement while on the medication.

## 3 Discussion

Pulmonary arterial hypertension (PAH) is a progressive disease of the lung vascular system, which can be idiopathic, heritable, or associated with other medical conditions such as congenital heart disease [19]. Heritable causes include mutations in bone morphogenetic protein receptor type 2 (BMPR2), endoglin (ENG), and activin-like kinase-type 1 (ALK1); the latter two mutations are associated with type 1 and type 2 HHT, respectively. This case report describes a patient with congenital heart disease (sinus venosus ASD and PAPVR) and type 1 HHT, who presented with severe and progressive PAH. After exhausting conventional therapeutic options for PAH, the patient was treated with sotatercept, a novel drug acting as a ligand trap for members of the transforming growth factor-beta superfamily. To our knowledge, this case report is the first to detail the clinical response of a patient with PAH and HHT to sotatercept therapy.

Sotatercept is a novel therapy that has shown promise in the treatment of PAH. The drug demonstrated significant efficacy in improving pulmonary hemodynamics in both the Phase 2 PULSAR and Phase 3 STELLAR trials [16]. While sotatercept has shown beneficial effects in the general PAH population, its role in patients with HHT remains unclear. There are no data regarding the use of sotatercept specifically in HHT-associated PAH, and the safety profile of sotatercept in HHT patients has not been established. Adverse events reported with sotatercept include telangiectasia, bleeding events, and thrombocytopenia [16]. These events are particularly concerning in individuals with HHT, who almost invariably suffer from bleeding related to mucosal telangiectasias in the nose or gastrointestinal tract. Our patient experienced a notable improvement in functional status after initiating sotatercept, though she did experience worsening of epistaxis. In patients who develop epistaxis, management strategies include nasal humidification, topical emollients, hemostatic nasal plugs, oral tranexamic acid, and ENT referral for cauterization or sclerotherapy. These supportive measures should be combined with monitoring of hemoglobin, platelet counts, and epistaxis severity, as bleeding events often occur within the first weeks of therapy. Thus far, no biomarkers have been shown to predict the worsening of bleeding during sotatercept therapy.

The patient has a complex medical history, including both genetically-confirmed HHT and a history of congenital heart disease (sinus venosus ASD and PAPVR), and the cause of PAH in her case may be multifactorial. Interestingly, the patient has type 1 HHT, which is only rarely associated with PAH; PAH is more commonly associated with type 2 HHT *ALK1* mutations [20].

This case underscores the complexity of managing PAH in patients with underlying HHT, and highlights the need for careful monitoring and individualized treatment strategies. Therapies such as sotatercept that can promote bleeding or telangiectasias should be approached with caution, but as illustrated in this case, the benefits may outweigh the risks. Further studies, which could include larger retrospective case series, prospective trials, or subgroup analyses within broader PAH studies, are needed to better understand how this promising therapeutic agent can be used in HHT patients with PAH.

## CRediT authorship contribution statement

**Abinaya Ramakrishnan:** Writing – review & editing, Writing – original draft, Resources, Formal analysis, Data curation. **Scott Olitsky:** Writing – review & editing, Writing – original draft, Supervision, Project administration. **Rajan Saggar:** Writing – review & editing. **Richard N. Channick:** Writing – review & editing. **Justin P. McWilliams:** Writing – review & editing, Writing – original draft, Supervision, Resources, Project administration, Methodology, Investigation, Formal analysis, Data curation, Conceptualization.

## Grants and financial support

None.

## Declaration of competing interest

The authors declare that they have no known competing financial interests or personal relationships that could have appeared to influence the work reported in this paper.
